# 454 screening of individual MHC variation in an endemic island passerine

**DOI:** 10.1007/s00251-014-0822-1

**Published:** 2014-12-18

**Authors:** Catalina Gonzalez-Quevedo, Karl P. Phillips, Lewis G. Spurgin, David S. Richardson

**Affiliations:** 1 School of Biological Sciences, University of East Anglia, Norwich Research Park, Norwich, NR4 7TJ UK; 2 Institute of Environmental Biology, Faculty of Biology, Adam Mickiewicz University, Poznań, Poland; 3 Behavioural Ecology and Self-organization, Centre for Ecological and Evolutionary Studies, University of Groningen, Groningen, Netherlands

**Keywords:** 454, Allele amplification efficiency, *Anthus berthelotii*, *Anthus campestris*, Berthelot’s pipit, Major histocompatibility complex, Tawny pipit

## Abstract

Genes of the major histocompatibility complex (MHC) code for receptors that are central to the adaptive immune response of vertebrates. These genes are therefore important genetic markers with which to study adaptive genetic variation in the wild. Next-generation sequencing (NGS) has increasingly been used in the last decade to genotype the MHC. However, NGS methods are highly prone to sequencing errors, and although several methodologies have been proposed to deal with this, until recently there have been no standard guidelines for the validation of putative MHC alleles. In this study, we used the 454 NGS platform to screen MHC class I exon 3 variation in a population of the island endemic Berthelot’s pipit (*Anthus berthelotii*). We were able to characterise MHC genotypes across 309 individuals with high levels of repeatability. We were also able to determine alleles that had low amplification efficiencies, whose identification within individuals may thus be less reliable. At the population level we found lower levels of MHC diversity in Berthelot’s pipit than in its widespread continental sister species the tawny pipit (*Anthus campestris*), and observed trans-species polymorphism. Using the sequence data, we identified signatures of gene conversion and evidence of maintenance of functionally divergent alleles in Berthelot’s pipit. We also detected positive selection at 10 codons. The present study therefore shows that we have an efficient method for screening individual MHC variation across large datasets in Berthelot’s pipit, and provides data that can be used in future studies investigating spatio-temporal patterns and scales of selection on the MHC.

**Electronic supplementary material:**

The online version of this article (doi:10.1007/s00251-014-0822-1) contains supplementary material, which is available to authorized users.

## Introduction

Genes of the major histocompatibility complex display the highest levels of genetic variation found in vertebrates (Bodmer et al. 1997; Torimiro et al. 2006; Mona et al. 2008), with some loci, like the human HLA-B, having more than 2,000 alleles (Robinson et al. 2013). Variants at the MHC are created by point mutation (Hughes and Nei 1988) and by gene conversion-like processes (Ohta 1995; also known as microrecombination; Geliebter and Nathenson 1988; hereafter, referred to as ‘gene conversion’; Edwards and Hedrick 1998; Spurgin et al. 2011). Pathogen-mediated selection—linked to the MHC’s central role in initiating immune reactions to pathogen antigens—is considered responsible for maintaining the high levels of individual and population variation observed at these loci, although sexual selection (reviewed in Edwards and Hedrick 1998) and other mechanisms may also play a role (van Oosterhout 2009).

To understand MHC variation, one must understand how it works: this family of duplicated genes codes for molecules that detect pathogens and initiate the adaptive immune response in vertebrates (Wakelin 1996; Wakelin and Apanius 1997). There are two main classes of MHC genes: class I codes for molecules that generally present peptides originated from proteins located in the cytoplasm, whereas class II codes for molecules that generally present peptides derived from proteins located in intracellular vesicles (Frank 2002). In class I MHC, exons 2 and 3 encode the peptide binding region (PBR, Bjorkman et al. 1987). The spatial configuration of folds and pockets in the PBR allows each MHC molecule to bind a specific range of peptides (Chelvanayagam 1996). Therefore, within- individual amino acid polymorphisms in the PBR of these MHC genes determine the number of peptides that can be recognized by a cell, and thus the number and type of pathogens an individual can defend itself against (Potts and Wakeland 1990). However, not all the different variants within MHC genes will generate molecules that bind different sets of pathogen peptides. It has been suggested that MHC variants may be defined into functionally distinct ‘supertypes’, grouping variants which encode for different sequences of amino acids which, nevertheless, have similar chemical and physical properties and thus similar binding specificities (Sidney et al. 1995). While the role of supertypes in pathogen recognition is not yet clear, some ecological studies suggest that statistical associations between pathogens and MHC variation occur at the supertype level (e.g. Schwensow et al. 2007; Sepil et al. 2013). Despite this last proviso, it is clear that MHC diversity is important, in terms of the ability to defend against pathogens both at the individual and population level (Meyer-Lucht et al. 2010; May et al. 2011).

The characterization of functional MHC alleles and correct assignment of individual genotypes are imperative for understanding patterns of adaptive variation in and among wild populations, for studying host-pathogen co-evolution (Klein et al. 1994; Sommer 2005), and potentially, for informing conservation where maximising such variation may be key to population persistence (Ujvari and Belov 2011; Wright et al. 2014). However, the presence of multiple gene copies and the sequence similarity among them (Kelley et al. 2005; Cheng et al. 2012) makes it difficult to design locus-specific primers, leading to co-amplification of alleles from multiple loci. Traditionally, MHC genotyping has been done by cloning and subsequent sequencing (e.g. Jarvi et al. 2004; Alcaide et al. 2008)—a process that is time-consuming, especially when applied to ecological-scale datasets. Other methods rely on conformational shifts between different alleles of the MHC which can be separated by gel electrophoresis (Mwenda et al. 1997; Baquero et al. 2006; Worley et al. 2008), but these methods are still time-consuming, and, unless they include the direct cloning/sequencing of identified variants, they cannot provide direct sequence information (Promerová et al. 2012).

With the introduction of next-generation sequencing (NGS) technologies, such as the Roche 454 pyrosequencing platform (Margulies et al. 2005), it is now possible to obtain sequences from individual DNA strands, allowing rapid and efficient parallel sequencing of co-amplified alleles. Another advantage of using NGS is the potential for obtaining sequences from a large number of identified individuals in a single run by using ‘barcoded’ primers. This allows for the subsequent assignment of sequences to individuals during the sequence processing step. Since its introduction in 2005, 454 has been widely used to sequence the MHC in a variety of organisms (e.g. Kloch et al. 2010; Sepil et al. 2012; Dunn et al. 2013). However, NGS techniques are not error free—for example, 454 sequencing is prone to errors, such as insertions, deletions and chimeras generated during the two required amplification steps (Meyerhans et al. 1990; Bradley and Hillis 1997) and during the pyrosequencing reaction and base calling (Huse et al. 2007; Beuf et al. 2012). Consequently, for accurate MHC genotyping, it is crucial to distinguish artefacts from real alleles (Galan et al. 2010; Sommer et al. 2013). Different methods have been proposed to detect sequencing artefacts (Babik et al. 2009; Galan et al. 2010; Promerová et al. 2012; reviewed in Lighten et al. 2014a), and these methods generally rely on thresholds of number of reads that a genuine allele is expected to be represented by. However, a problem that these approaches do not address is that alleles differ in their amplification efficiency, meaning that some alleles will be systematically missed from individuals where they are present (Sommer et al. 2013). This ‘allelic dropout’ can inflate homozygosities and deflate individual MHC diversity if it is not accounted for (Sommer et al. 2013).

Berthelot’s pipit (*Anthus berthelotii*) is a sedentary passerine endemic to 12 islands across the Canary, Madiera and Salvagens archipelagos in the Macaronesian region (Cramp and Perrins 1977, Fig. 1). Previous work has shown that the population bottlenecks that occurred during the colonization of each archipelago (Illera et al. 2007; Spurgin et al. 2014) substantially reduced MHC variation in this species (Spurgin et al. 2011). However, MHC variation has, at least partially, been regenerated largely by gene conversion (Spurgin et al. 2011). Interestingly, the pipit populations are exposed to consistent but spatially varying pathogen pressures both within (Gonzalez-Quevedo et al. 2014) and among populations (Spurgin et al. 2012). Thus these populations provide an excellent system in which to test different evolutionary hypothesis on the role of pathogen-mediated selection in shaping the patterns of MHC variation at various spatio-temporal scales. Population-level variation at the MHC of Berthelot’s pipit has been assessed (Spurgin et al. 2011), but individual level screening within populations is needed if we are to investigate the factors that drive MHC variation at different scales. With that as our overall aim, here we test the utility of the methods outlined by Sommer et al. (2013) to individually sequence MHC class I exon 3 variation in 310 Berthelot’s pipits from Tenerife and from 10 tawny pipits (*Anthus campestris*), a geographically widespread species that is the closest relative of Berthelot’s pipit (Voelker 1999). Using the data generated, we then compare the levels of MHC variation found in the two species, and test for signatures of selection.

**Fig. 1:**
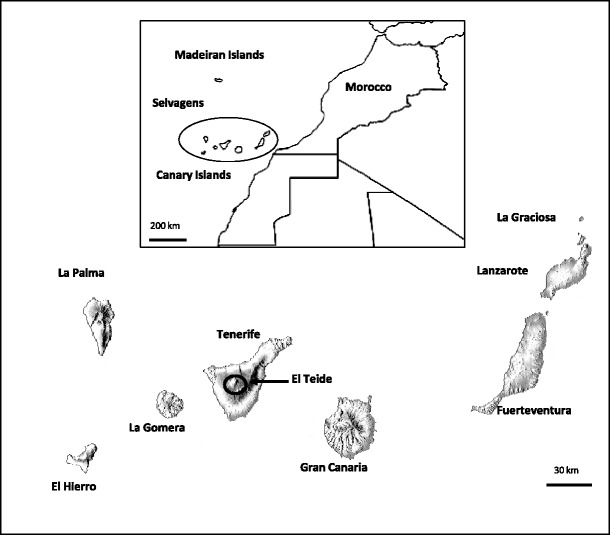
Distribution of Berthelot’s pipits (*Anthus berthelotii*) in the Macaronesian archipelagos of Madeira, Selvagens and the Canary Islands (*inset*), and detail of the nine populations from the Canary Islands

## Materials and methods

### Study species and sampling

We sampled Berthelot’s pipits on Tenerife, in the Canary Islands, from January to April 2011. To obtain a representative sample across the pipit range on Tenerife, a 1km^2^ grid was laid over a map of the island obtained from Google Earth in ArcGIS version 10 (Esri 2011, Redlands, CA, www.esri.com). Most accessible square kilometres that contained habitat suitable for pipits were visited and, where present, an attempt was made to catch at least one pipit per km^2^ using clap nets baited with *Tenebrio molitor* larvae. Each captured bird was fitted with a unique metal ring from the Spanish Environment Ministry and a ca. 25μl blood sample was taken by brachial venipuncture and stored in absolute ethanol in a 2ml screw cap micro-centrifuge tube at room temperature. The final sample of 388 birds included 30 birds caught on the mountain of El Teide on Tenerife (2,500 m above sea level), a separate population which is isolated from the lowlands by dense pine and laurel forest which the pipit does not inhabit (Illera 2007). Ten tawny pipits (two from Morocco, three from Mauritania and five from the Iberian Peninsula) were also screened.

### MHC genotyping

DNA was extracted from blood using a salt method (Richardson et al. 2001). In line with previous studies on passerines (e.g. Westerdahl et al. 2005; Bonneaud et al. 2006; Alcaide et al. 2013) and on Berthelot’s pipit (Spurgin et al. 2011), and because of the resources available to us, we sequenced only exon 3 of MHC class I. Pooling large numbers of individuals in a single 454 run can result in highly uneven representation of individuals (S. Paterson, personal communication), thus samples were pooled in eight pools of 80 samples each. In total, 310 Berthelot’s pipits (including 26 individuals from El Teide) out of the 388 originally sampled, and 10 tawny pipits were genotyped at the MHC. We screened each of the 320 samples twice, and each replicate of a given sample was amplified using different combinations of barcoded primers (thus screening a total of 640 amplicons). For the library preparation, we used forward and reverse fusion primers consisting of the 454 adaptor (forward adaptor, 5ʹ-CGTATCGCCTCCCTCGCGCCA-3ʹ and reverse adaptor, 5ʹ-CTATGCGCCTTGCCAGCCCGC-3ʹ), followed by a key sequence (TCAG), a 10-bp multiplex identifier (MID), and the MHC class I exon 3 specific primers (DG2, 5ʹ-TTGCGCTCYAGCTCYTTCTGCT-3ʹ and GENDG, 5ʹ-TCCCCACAGGTCTCCACAC-3ʹ; Spurgin et al. (2011), Fig. S1). MID sequences were obtained from the 10-base extended MID set from Roche Diagnostics (454 Life Sciences Corp. 2009). We chose nine MIDs with at least three base pair differences between them (MID numbers 1, 2, 3, 4, 5, 7, 8, 10 and 11). By using these MIDs in the forward and reverse primers, we had 81 possible combinations with which to barcode individuals within the pools of 80 samples. We pooled samples so that the two replicates of a sample were present in different pools and had a different forward and reverse fusion primer combination. Two strategies were implemented in order to reduce the formation of chimeras during the PCR (Lenz and Becker 2008; Holcomb et al. 2014). First, each of the 640 amplicons screened was generated by two independent PCRs that were then pooled in equimolar amounts; second, the number of cycles was reduced to the minimum number that provided a clear, well-defined amplicon when visualised in an agarose gel (27 cycles). PCRs were performed in 25 μl volumes containing 0.5 μM of each fusion primer, 12.5 μl of 2× Roche FastStart master mix and ca. 60 ng of DNA. Thermocycling consisted of an initial denaturation at 96 °C for 4 mins, followed by 27 cycles of 94 °C for 30 s, 61 °C for 30 s and 72 °C for 60 s, with a final extension of 72 °C for 10 mins. PCR products were run on 1.5 % agarose gels, and amplicons were cut from the gels using a sterile scalpel and purified using a gel purification kit (Qiagen). Purified products were quantified using the PicoGreen dsDNA assay kit (Life technologies) and pooled in order to get 1.25 ng per sample for each pool of 80 samples. Emulsion PCR and 454 sequencing were conducted at the Centre for Genomic Research at the University of Liverpool. Sequencing was run on a PicoTitre plate (each pool in one eighth of the plate) on a GS FLX Titanium system.

### Bioinformatics

High-quality sequences (Phred quality score > 20 at more than 95 % bases) with complete forward and reverse MID and primer sequences were assigned to amplicons based on MID combinations. We then followed the workflow outlined by Sommer et al. (2013) to assign reads to putative MHC alleles and to identify artefacts based on the assumptions that: (1) artefacts are less frequent than their source allele, and (2) artefacts should have a lower intra-amplicon frequency than any true allele. The workflow consists of three main steps that classify variants into ‘putative alleles’, ‘putative artefacts’, and ‘unclassified variants’. In the first step, applied to each amplicon independently, reads were assigned to groups of identical sequences, hereafter referred to as ‘clusters’. The most frequent cluster within each amplicon was classified as a putative allele. Sequences represented by only one read were discarded as sequencing artefacts. Clusters were then classified as ‘chimera’, ‘1–2 bp difference’ or ‘>2 bp difference’ by comparing their sequences to more frequent clusters within the amplicon (full details in Sommer et al. 2013). Chimeras were identified using a Python script that checked within each amplicon whether a given sequence could, at any point along its length, be formed by joining together the forward section of a more frequent sequence with the reverse section of another more frequent sequence (further details in Appendix 1, supplementary information).

The second step of the pipeline compares both replicates of a given individual. Clusters listed as ‘chimera’ or ‘1–2 bp difference’ in step one were classified as putative artefacts if they were absent from the replicate amplicon. Clusters were also classified as putative artefacts if they had been identified as chimeras in both replicates. Clusters classified as ‘>2 bp difference’ were only classified as putative artefacts if, across the whole data set, they were unique to a single amplicon. All other clusters were retained for further checking in step three.

In step three, clusters retained on the ‘1–2 bp difference’ list from step two (i.e. those that were observed in both replicate amplicons) were classified as putative alleles if they had a higher intra-amplicon frequency than any entry in the putative artefact list, and as unclassified variants if this criterion was not met. The same process was used on clusters in the ‘>2 bp difference’ list when the sequence was also present in the sample’s replicate. Other clusters in the ‘>2 bp difference’ list were labelled as unclassified variants if present as a putative allele in another individual, but as putative artefacts if not. Chimeras retained from step two were classified as putative alleles if the same sequence was present as a putative allele in another individual; otherwise, retained chimeras were labelled as unclassified variants.

After completing the pipeline, we further checked the lists of putative alleles, putative artefacts and unclassified variants for Berthelot’s and tawny pipits separately. For Berthelot’s pipits, we discarded sequences classified as putative alleles that occurred in only one individual, but pulled out sequences from the unclassified variants list that matched sequences in the list of putative alleles. We assessed the frequency of each unclassified variant in the amplified samples (the number of individuals they occurred in out of the total of 310), and further inspected for the presence of any MHC alleles that had been described in the previous, population-level characterisation of MHC variation in Berthelot’s pipits (Spurgin et al. 2011). We also inspected the sequences in the putative artefacts list by pulling out sequences that matched a putative allele already identified in another individual, and that were also present in both amplicons of a bird with an intra-amplicon frequency higher than the least frequent entry to the list of putative alleles.

For tawny pipits, we proceeded differently due to the small sample size. If a variant was classified as a putative allele in both replicates of only one sample, it was kept on the putative alleles list. If the variant was classified as an allele in only one amplicon, but was found in other amplicons as an unclassified variant, it was also treated as a putative allele. Finally, for unclassified variants, we pulled out and classified as alleles sequences that were present in both replicates of at least two birds. For both species, repeatability of genotyping was calculated as the percentage of shared alleles between the two replicate amplicons of each individual.

### Allele amplification efficiency

We followed Sommer et al.’s (2013) rationale, which assumes that the amplification efficiency of an allele is independent of the genotype and similar among PCR products with the same conditions, to estimate the relative amplification efficiency of each allele. We used scripts provided in Sommer et al. (2013) to perform this calculation in R (R Development Core Team 2011). We standardised allele amplification efficiencies relative to ANBE11 and ANCA17 for Berthelot’s and tawny pipits, respectively. The choice of standardising alleles is arbitrary, as our use of degenerate primers means we cannot know which allele is the ‘best’ amplifier in our data set. We also calculated a variant of Galan’s T1 (Galan et al. 2010), which uses the lowest amplification efficiency to estimate the minimum number of reads per amplicon necessary to reach a coverage of 99.9 % for a genotype with a given number of alleles (in our case, 12—the maximum number of alleles observed in an individual) and with a minimum number of two reads per allele. This calculation was done using the R function ‘T1.min.efficiency.replicated’ provided in Sommer et al. (2013). Any sample that had a number of reads lower than the T1 threshold was discarded and not used in downstream analyses.

### MHC sequence analyses

Using the software DnaSP 5.10.01 (Librado and Rozas 2009), we calculated the number of nucleotide differences and nucleotide diversity among sequences for the set of alleles identified in each species. In order to investigate the mutation to recombination ratio among MHC alleles, we calculated the recombination (*R*_m_) and mutation (*Ɵ*) parameters (Hudson and Kaplan 1985) and obtained the 95 % confidence interval using a coalescent approach with 10,000 replications. To compare allele diversity between the two species we calculated pairwise nucleotide distance using the Nei-Gojobori/Jukes-Cantor method (Nei and Gojobori 1986) in MEGA 6 (Tamura et al. 2011), and assessed differences using a Mann–Whitney U test.

To explore phylogenetic relationships among Berthelot’s and tawny pipit MHC class I alleles we built a neighbour net with Jukes-Cantor distance between all pairs of alleles using the software SplitsTree 4.13.1 (Huson and Bryant 2006). We explored the presence of gene conversion tracts in the MHC class I alleles identified using the following methods: 3Seq (Boni et al. 2007), GENECONV (Padidam et al. 1999), MaxChi (Smith 1992), Chimaera (Posada and Crandall 2001) and SiScan (Gibbs et al. 2000), all implemented in the software RDP4 (Martin et al. 2010). The highest acceptable *P* value was set to 0.05, and 100 permutations were performed for all methods. Tracts identified by at least two methods were considered true recombination events.

The number of putative functionally different MHC class I alleles was estimated based on the amino acid sequences. The codons involved in the peptide binding region (PBR) of the MHC class I of Berthelot’s pipits have been identified previously (Spurgin et al. 2011) based on the sites known to code for the PBR in humans (Brown et al. 1993). Using the Berthelot’s pipit sequences, the rate of non-synonymous (*d*_N_) and synonymous (*d*_S_) substitutions per site was calculated in MEGA 6 (Tamura et al. 2011) using the Nei-Gojobori/Jukes-Cantor method (Nei and Gojobori 1986) for, (1) the full exon sequence, (2) the non-peptide binding region (non-PBR), and (3) the peptide binding region (PBR). Differences between *d*_N_ and *d*_S_ were assessed with Mann–Whitney *U* tests. We did not perform these analyses on tawny pipits because the small sample size for this species means that we have likely underestimated the number of alleles.

In order to identify codon-specific signatures of positive selection at the MHC class I across the two pipit species, four codon-based methods to detect selection based on the *d*_N_ and *d*_S_ were implemented in the webserver Datamonkey (http://datamonkey.org, Pond and Frost 2005a). The fixed effects likelihood (FEL), random effects likelihood (REL, Pond and Frost 2005b), and fast unbiased Bayesian approximation (FUBAR, Murrell et al. 2013) were used to detect codons under pervasive selection. In addition, the mixed effects model of evolution (MEME, Murrell et al. 2012) was used to detect codons under episodic diversifying selection. Sites with Bayes factor > 50 for REL, posterior probabilities > 0.9 for FUBAR and *P* values < 0.1 for FEL and MEME were considered to have enough support for positive selection. Only sites that were detected to be under positive selection by at least two different methods were considered to be candidates of evolution under positive selection. Prior to running analyses, the best fitting nucleotide substitution model was determined using a model selection approach also implemented in Datamonkey (http://datamonkey.org, Pond and Frost 2005a). All sequences identified from both Berthelot’s and tawny pipits were used in this analysis.

We explored whether MHC alleles found in Berthelot’s pipits could be clustered into ‘supertypes’ (Doytchinova and Flower 2005) according to the antigen-binding characteristics of either the amino acids in the PBR (15 amino acids), or the amino acids that were detected as positively selected sites (PSS) by our analysis. Five descriptors were obtained for each amino acid (Sandberg et al. 1998): z1 (hydrophobicity), z2 (steric bulk), z3 (polarity), z4 and z5 (electronic effects). Amino acid descriptors were arranged in a matrix where each row represented one unique PBR or PSS sequence and the columns represented the five descriptors for each amino acid in the region being analyzed. We performed a *k*-means clustering algorithm to identify the most likely number of clusters of alleles based on the amino acid descriptors using the function ‘find.clusters’ in the ‘adegenet’ package (Jombart 2008; Jombart et al. 2010) in R. The algorithm was run four times for different numbers of clusters from one to the total number of unique sequences, and for each run a mean Bayesian information criterion (BIC) was obtained. The most likely number of clusters in the data is the one with the lowest BIC. After identifying the optimal number of clusters, a discriminant analysis of principal components (DAPC) is used to identify the alleles in each cluster (Jombart et al. 2010).

## Results

### MHC allele identification

We obtained a total of 1,019,897 high-quality sequences ranging from 53 to 4,308 reads per amplicon (mean ± S.D. = 1436 ± 652). Of these, 919,046 reads had complete forward and reverse MIDs and correct primer sequences, leaving 45–4,049 reads per amplicon (1427 ± 647) and 744–6,263 reads per individual (2855 ± 954).

At the end of the bioinformatics processing, 41 clusters were classified as putative alleles, of which 31 were assigned to Berthelot’s pipits and 10 to tawny pipits. The list of unclassified variants contained the greatest number of entries, followed by putative artefacts and putative alleles (Table S1). We discarded 11 of the 31 putative alleles detected in Berthelot’s pipits because they were classified as alleles in only one individual. Since these variants were not confirmed across individuals, we believe these must be either artefacts or very low frequency alleles. We determined that the more conservative approach would be to discard them, as even if they were true alleles, at such low frequencies (ca. 0.3 %) they would have little consequence in downstream analyses. We identified 588 clusters as unclassified variants, of which 134 were present in more than two samples. Among these clusters we found two—ANBE3 and ANBE31—that occurred in both amplicons of more than 80 % of birds and were considerably more frequent in the 310 Berthelot’s pipit samples than all the other unclassified variants (Fig. S2), but had a very small number of reads in most amplicons (mean ± S.E.: ANBE3 = 24.5 ± 0.8; ANBE31 = 18.0 ± 0.6). These two variants also matched sequences identified by Spurgin et al.’s (2011) previous population-level study on MHC in Berthelot’s pipit. We treated ANBE3 and ANBE31 as ‘low efficiency alleles’ (see amplification efficiency results below), but because we could not be certain of their absence/presence in all individuals we recommend they are excluded from future individual based MHC-disease association studies in Berthelot’s pipit. However, we retained these alleles when assessing sequence-level selection, as their amplification efficiency should not bias such analyses.

After processing the unclassified variants, we were left with 22 alleles identified for the Tenerife population of Berthelot’s pipits. Of these, seven (named ANBE43-ANBE49) had not been previously identified in this species, and have been deposited in GenBank (accession numbers KM593305–KM593311). We also detected seven alleles (ANBE1, ANBE6, ANBE7, ANBE9, ANBE13, ANBE31 and ANBE38) that Spurgin et al. (2011) found in other populations of Berthelot’s pipits but not in Tenerife. Among these, ANBE31 had previously been found only on Lanzarote (another Canary Island), and ANBE38 had only been found on Selvagem Grande (one of the islands of the Selvagens archipelago). We failed to find five alleles that had been previously reported from an earlier, smaller sample of birds (30 individuals sampled in 2006) from each of the Tenerife and Teide populations (Spurgin et al. 2011): ANBE12 had been detected previously on El Teide, and Fuerteventura (one of the Canary Islands); ANBE24 had been detected previously in the low lands of Tenerife and in other islands of the Canary archipelago; ANBE19 and ANBE39 had been previously detected in the low lands of Tenerife and on El Teide, and ANBE41 was restricted to the population of El Teide.

**Fig. 2:**
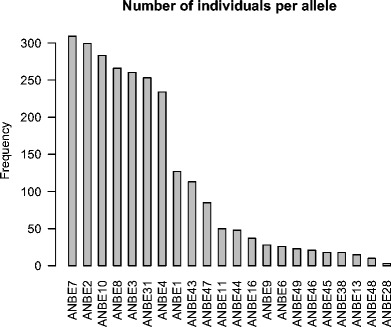
Frequency of Berthelot’s pipit (*Anthus berthelotii*) MHC class I, exon 3 alleles identified in 310 individuals in the population on Tenerife

Intra-amplicon frequencies (proportion of reads within an amplicon) of the 22 alleles identified in Berthelot’s pipits varied from a low of 0.013 for ANBE31 (S.E. = 0.0003) to a high of 0.192 for ANBE10 (S.E. = 0.003, Fig. S3a). Population allele frequencies ranged from 0.01 for ANBE28 to 0.99 for ANBE7 (Fig. 2). The number of alleles per individual ranged from 4 to 12, with a mode and median of eight alleles (Fig. S4), suggesting the potential presence of six loci in this species.

**Fig. 3:**
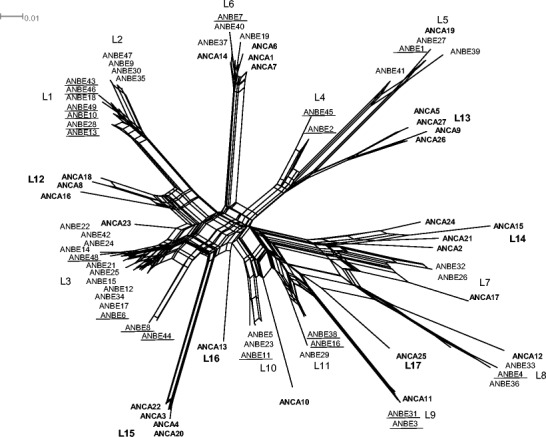
Neighbour net of 49 Berthelot’s pipit (*Anthus berthelotii*, ANBE) and 27 tawny pipit (*A. campestris*, ANCA) MHC class I, exon 3 alleles using Jukes-Cantor distance, including alleles previously identified in Berthelot’s pipits (Spurgin et al. 2011). ANBE alleles that we found in the present study are *underlined*. Alleles and lineages found exclusively in tawny pipits are shown in *bold font*. Labels L1–L17 correspond to lineage names. *Length along lines* is proportional to genetic distance between any two alleles

After processing unclassified variants for the tawny pipit we were left with 28 clusters, of which 11 were classified as putative alleles and 17 as unclassified variants. One allele, ANBE9, was shared between Berthelot’s and tawny pipits. The other 27 alleles, unique to the tawny pipit, were named ANCA1 to ANCA27 and their sequences have been deposited in GenBank (accession numbers KM593312–KM593338). Intra-amplicon frequencies for these 28 alleles ranged from a low of 0.015 (S.E. = 0.003) for ANCA20 to a high of 0.142 (S.E. = 0.039) for ANCA1 (Fig. S3b). The number of alleles per individual in tawny pipits ranged from 6 to 11 (mean = 7.4, median = 7, Fig. S4), which also suggests the presence of six loci.

The repeatability (i.e. the mean percentage of alleles shared between the two replicates of the same sample) of our genotyping was 96.1 %. (S.E. = 5.45). The lowest repeatability (44.4 %) was obtained for a sample with an amplicon with only 45 reads. Fourteen samples had repeatabilities lower than 80 % and 240 samples had a repeatability of 100 %.

### Allele amplification efficiencies

In Berthelot’s pipits, the lowest amplification efficiency was obtained for allele ANBE31 (0.2, i.e. five times lower than the reference ANBE11). The highest amplification efficiency obtained was for ANBE10 (3.0) (Fig. S5). The modified Galan’s T1 threshold showed that 139 reads (range 132–144) were needed to reach coverage of 99.9 % of a genotype with twelve alleles (the highest possible given the number of loci estimated). Given this value, we excluded the one sample (identified above) that had only 45 reads in one of its replicates from all downstream analyses. In tawny pipits, the lowest amplification efficiency obtained was for ANCA20 (0.33) and the highest amplification efficiency for ANCA1 (3.9) (Fig. S5). The modified Galan’s T1 threshold for the lowest amplification efficiency in tawny pipit alleles was 291. All tawny pipit amplicons had more than 291 reads.

### MHC sequence analyses

Descriptors of sequence variation within MHC class I exon 3 in Berthelot’s and tawny pipits are summarised in Table 1. Seven alleles (ANBE3, ANBE31, ANCA11, ANCA12, ANCA13, ANCA17 and ANCA25) had an insertion of three nucleotides, resulting in a protein with one amino acid insertion but no disruption of the reading frame. Pairwise nucleotide distance of MHC alleles was significantly higher in tawny pipit than in Berthelot’s pipit (*P* < 0.001, Table 1).

**Table 1:** Summary of nucleotide variation of MHC class I, exon 3 sequences identified in this study of Berthelot’s pipits, *Anthus berthelotii*, and tawny pipits, *A. campestris*

Descriptor	Berthelot’s pipit	Tawny pipit
Number of alleles	20	28
Number of variable sites	84	103
Number of mutations	105	136
*π* ± SE^a^	0.11 ± 0.01	0.14 ± 0.01
*k* ± SE^b^	27.09 ± 0.86	32.84 ± 0.81
*R*_m_ (95 % CI)^c^	32.5 (25.0–40.0)	66.7 (52.0–83.1)
*Ɵ* (95 % CI)^d^	23.0 (19.2–27)	32.9 (27.2–38.5)

^a^Nuclotide diversity ± standard deviation

^b^Average number of nucleotide differences ± standard deviation

^c^Recombination rate (lower and upper 95 % confidence limits)

^d^Mutation rate (lower and upper 95 % confidence limits)

The neighbour net of Berthelot’s and tawny pipits MHC class I exon 3 alleles revealed a total of 17 lineages (Fig. 3). The lineage partitions were chosen based on the previous phylogeny described for Berthelot’s pipit MHC class I alleles (Spurgin et al. 2011). Berthelot’s pipit alleles were included in eleven lineages, while tawny pipit alleles were grouped in 13 lineages, of which seven were shared with Berthelot’s pipit and six were unique to tawny pipits (Fig. 2). The presence of boxes in the net means that there can be several paths between any two alleles, typical of sequence datasets with gene conversion and recombination events (Bryant and Moulton 2004). Four gene conversion events in MHC class I alleles of Berthelot’s and tawny pipits were identified by at least two methods (Table 2).

**Table 2:** Tracts of gene conversion identified by at least two recombination-detection methods in MHC class I alleles of Berthelot’s pipits (*Anthus berthelotii*, ANBE) and tawny pipits (*A. campestris*, ANCA). Position corresponds to nucleotides that limit the maximal gene conversion tract

Recombinant allele	Major parent	Minor parent	Positions of breakpoints	Methods (*P* value)
ANBE4	ANCA18	ANCA5	35–179	MaxChi (0.014) 3Seq (0.039)
ANCA16	ANCA13	ANBE6	48–216	SiScan (0.037) 3Seq (0.001)
ANCA17	ANCA15	ANBE44	170–238	Chimaera (0.005) 3Seq (0.003)
ANCA12	ANCA18	ANCA5	12–197	MaxChi (0.014) 3Seq (0.039)

The 22 Berthelot’s pipit MHC class I alleles translated as 20 different amino acid sequences, containing 42 variable amino acid sites and 63 amino acid changes (14 positions had more than two amino acids, Fig. S6). Considering only the 15 PBR sites, 15 unique PBR sequences were detected, encoded by 12 variable amino acids (80 %, Fig. S7a), while the 65 non-PBR sites harboured 30 variable amino acids (46 %). The 28 MHC class I alleles found in tawny pipits translated as 24 unique amino acid sequences containing 47 variable amino acid sites, with 86 amino acid changes (18 positions had more than 2 amino acids, Fig. S6). Considering only the PBR sites, there were 20 unique PBR sequences, with 12 variable amino acids (80 %), while the 65 non-PBR sites had 36 variable amino acids (55 %, Fig. S7b).

**Fig. 4:**
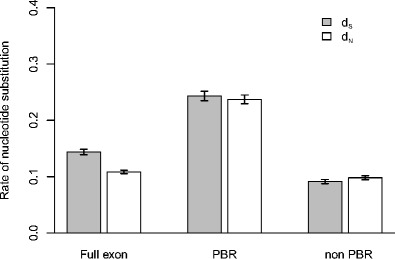
Rates of non-synonymous (*d*_N_) and synonymous (*d*_S_) substitutions in the full exon, peptide binding (PBR) and non-PBR regions MHC class I exon 3 alleles of Berthelot’s pipits in the population on Tenerife. *Error bars* represent 95 % confidence interval

Over the full exon 3 sequence of Berthelot’s pipit MHC class I, the rate of synonymous substitutions (*d*_S_) was significantly higher than the rate of non-synonymous substitutions (*d*_N_) (*P* < 0.001, Fig. 4), and the ratio of *d*_N_/*d*_S_ for the full exon was 0.75. In both the PBR and non-PBR regions, *d*_N_ was not significantly different from *d*_S_ (*P* = 0.45 and 0.52, respectively). Considering only the PBR *d*_N_/*d*_S_ = 1.07 and for the non-PBR *d*_N_/*d*_S_ = 0.97. However, both *d*_S_ and *d*_N_ were significantly higher at the PBR compared to the non-PBR (*P* < 0.001, Fig. 4). When performing selection tests with sequences identified in both pipit species, ten codons were identified as having evidence of positive selection. Four of these (5, 19, 61 and 62) occur in the PBR (Fig. S6).

The number of supertypes of Berthelot’s pipit MHC class I alleles that could be identified with the *k*-means clustering algorithm was 20, and therefore equal to the number of unique amino acid sequences identified. This was also the case if only alleles that differed at sites within the PBR (15 PBR unique sequences) and the PSS (11 PSS unique sequences) were included, suggesting that each allele has different antigen binding properties. For this reason, we did not perform the discriminant analysis of principal components (DAPC) to identify the alleles in each cluster (Jombart et al. 2010).

## Discussion

We used 454 pyrosequencing to screen MHC class I exon 3 variation in individual Berthelot’s pipits from across the population of Tenerife and, for comparison, in its sister species the tawny pipit. The variant/artefact identification procedure including replication of all samples allowed the successful genotyping of 309/310 Berthelot’s pipits, with high genotype repeatability (96 %). Validation procedures identified two alleles with low amplification efficiencies. The resulting sequence data were used to characterise MHC variation across the two closely related pipit species. We found significantly higher MHC sequence variation in the outbred tawny pipit than in the previously bottlenecked Berthelot’s pipit and found evidence for the maintenance of divergent MHC supertypes in Berthelot’s pipit. We also found evidence for gene conversion, an increased recombination to mutation ratio, and positive selection at specific codons within the exon 3 sequence.

454 pyrosequencing is a valuable tool for parallel sequencing of multilocus genes, such as those of the MHC, in a large number of samples. However, this method is prone to errors generated from two PCR amplifications and the pyrosequencing reaction (Meyerhans et al. 1990; Huse et al. 2007). In our study, artefacts and unclassified variants clearly outnumbered putative alleles. This concurs with other studies that used 454 for genotyping the MHC, which also assigned more reads to artefacts than to putative alleles (Zagalska-Neubauer et al. 2010; Sepil et al. 2012; Sommer et al. 2013).

For accurate reconstruction of individual MHC genotypes and testing of evolutionary hypotheses, it is essential to be able to reliably detect the majority of alleles that exist in a population, even if some of them do not amplify well in the study species. That MHC alleles differ in amplification efficiency has been known for some time (Babik 2010; Kiemnec-Tyburczy et al. 2010; Zagalska-Neubauer et al. 2010), but only recently has a methodology been proposed to calculate amplification efficiencies for each allele (Sommer et al. 2013). Of the 22 MHC alleles we identified in Berthelot’s pipit, two had low amplification efficiencies that put them at high risk of allelic dropout. These two alleles never amplified well enough to make the initial list of putative alleles, and were only identified by post-processing examination of the list of unclassified variants. Sommer et al. (2013) report 3/64 of the alleles they identified as having low amplification efficiency. This, together with our results, supports their hypothesis that low amplification is common but that it only affects a few alleles. However, it is important to note that some alleles might not amplify at all for a given set of primers, and that some instances of allelic dropout will thus not be accounted for. It is crucial that studies on the MHC acknowledge the variable amplification efficiency of alleles (regardless of the sequencing method used), and that they use the minimum amplification efficiency to obtain a minimum number of reads required per amplicon for reliable genotyping. When assessing MHC-disease associations, we advocate excluding samples that do not pass the minimum-read criterion, as well as excluding alleles with low amplification efficiency. Failure to do so incurs a high risk of incomplete data, i.e. missing the presence of these specific alleles within individuals, leading to inaccurate conclusions. We suggest that the calculation of amplification efficiencies be implemented and reported in all future MHC studies.

Our results differ to some degree from the earlier study that sequenced the MHC in the populations of Berthelot’s pipit (Spurgin et al. 2011). We found seven alleles that had not been described for Tenerife, but had been found in other islands. The smaller sample size of the earlier study (only 30 individuals from each of the populations in El Teide and Tenerife lowlands) might have been the reason why four of these were not detected (ANBE6, ANBE9, ANBE13 and ANBE38) because in the present study these were found at frequencies lower than 0.1. However, the other three alleles (ANBE1, ANBE7 and ANBE31) were found at frequencies of 0.4, 0.9 and 0.8, respectively in the present study, suggesting they are common in Tenerife. We also detected seven alleles that had not been described in any of the populations from the earlier study (ANBE43-ANBE49), and thus may be unique to the population of Tenerife. The frequencies of five of these alleles was lower than 0.1, thus not finding them previously was probably due to the small sample size of that earlier study. However, the other two alleles, ANBE43 and ANBE47, were found at frequencies of 0.4 and 0.3, respectively, and it is unclear why these were not detected in the earlier study. We also failed to find five alleles that had been previously reported in Tenerife (Spurgin et al. 2011): ANBE12 and ANBE39 from El Teide, ANBE41 and ANBE24 from the lowlands and ANBE19 from both El Teide and lowlands. In that earlier study, these alleles were not identified as putative ancestral alleles, but rather as recombinant alleles that were derived through gene conversion. These five recombinant alleles did not co-occur with their ancestral alleles on Tenerife, thus rejecting the possibility that they could have been PCR artefacts derived from the putative ancestral alleles (Spurgin et al. 2011). That we failed to detect alleles ANBE19, ANBE24 and ANBE41 could be due to the possibility that they are rare in lowland Tenerife. However, this is unlikely given that they were earlier detected in a much smaller sample (30 individuals), and given our sampling scheme that facilitated capture of variation throughout the population. However, in this study we could have reasonably missed ANBE12 and ANBE39 in the 26 birds we sampled from El Teide if they are rare in this population. Since the earlier study did not assess individual-level variation, it is not possible to know the frequency of these alleles in the earlier sample.

We cannot discard the possibility that the differences between the two studies lie in their methodological differences. The earlier study assessed MHC class I variation at the population level using population-specific tags, rather than individual-specific tags. Another difference is that here we sequenced only from the forward end of the amplicon, while the earlier study sequenced amplicons from both ends with subsequent assembly of sequences by read overlap. This is because, at the time of the earlier study, the average 454 read length was shorter than the total length of the amplicon. However, both studies used the same template-specific primer sequences and the same PCR conditions. The earlier study also used a different bioinformatics strategy for validating alleles. It is difficult to determine in which of the two studies errors were generated, given that both used different methodologies. It is also possible that the differences may be real and due to differences in the subset of individuals sampled in each case. For example, allele frequencies might have changed from 2006, when the samples of the earlier study (Spurgin et al. 2011) were collected, to 2011. Given that Berthelot’s pipit generation time is ca. 3 years (Garcia-Del-Rey and Cresswell 2007), approximately two generations have passed between the two samplings. In support of this hypothesis, rapid changes in MHC allele frequencies (between successive cohorts) have been reported in another passerine (Westerdahl et al. 2004). This pattern might result from fluctuating pathogen-mediated selection on these alleles. However, we cannot assess this with our data and further investigation involving sampling across different years is needed in order to confirm this.

It might be necessary to employ our individual-level MHC genotyping to screen the same samples that were used in the earlier study in order to clarify the source of the differences between the two studies. It is also important to note that other NGS technologies might provide better alternatives for accurately sequencing the MHC. For example, Illumina has proven to be highly repeatable and accurate for genotyping duplicated loci (Lighten et al. 2014b) and might therefore be the platform of choice in future studies that require sequencing of the MHC. However, there may equally be problems with other NGS methods, and only future comparative studies between methods will resolve such issues.

Maintaining and restoring MHC diversity is especially important in isolated, bottlenecked populations where low genetic diversity might have implications for disease resistance and population survival (Bollmer et al. 2011; Yasukochi et al. 2012; Niskanen et al. 2014). Isolated species with populations that have undergone bottlenecks generally show lower MHC genetic diversity than outbred species (reviewed in Radwan et al. 2010). In the present study 22 alleles were detected in the 309 individuals sampled in the Berthelot’s pipits population on Tenerife, compared to the 28 alleles detected in just 10 tawny pipits. This suggests that the Berthelot’s pipit population on Tenerife has much lower levels of genetic variation at these MHC loci than its continental sister species. This is confirmed by the fact that levels of nucleotide variation were lower in Berthelot’s pipit alleles than in tawny pipit alleles (nucleotide diversity = 0.11 ± 0.01 and 0.14 ± 0.01, respectively). However, if we consider the whole range of Berthelot’s pipits, a total of 49 MHC class I alleles have been identified across the 13 populations, indicating that MHC variation can be regenerated in a bottlenecked population, in this case by gene conversion (see below). The same pattern of higher genetic diversity in tawny pipit compared to Berthelot’s pipit has been described for the Toll-like receptor loci (TLR), of the innate immune system (González-Quevedo et al., The role of drift and selection in shaping variation at innate immune genes in oceanic island populations, unpublished).

Interestingly, we found that each Berthelot’s pipit MHC class I allele represented one supertype, suggesting that each allele has unique binding properties. In isolated island populations, such as those of Berthelot’s pipit, alleles may be lost during bottleneck events, but the maintenance of supertypes through these events may occur if alleles of different supertypes segregate at different loci and thus, PMS results in the maintenance of these functionally divergent alleles at different loci (van Oosterhout 2013). Our finding concurs with previous evidence for the maintenance of divergent supertypes in other systems (Huchard et al. 2008; Ellison et al. 2012). This process has important evolutionary implications in bottlenecked populations, allowing the maintenance of divergent MHC alleles that can potentially detect a broad range of pathogens.

Our neighbour net of the phylogenetic relationships among Berthelot’s and tawny pipit MHC class I alleles revealed that these species share allele lineages and do not separate according to species. Seven out of the 17 MHC lineages found were shared between Berthelot’s and tawny pipits and one allele (ANBE9) was found to be exactly the same across the 240 base pairs we screened in both species. Given the low sample size for the tawny pipit, it is possible that we missed amplifying some lineages and thus our estimate of lineage sharing must be treated as approximate. Furthermore, when colonizing the Canary Islands, some MHC lineages might have been absent from the founding population, hence explaining why some lineages found in tawny pipits are not represented in Berthelot’s pipits. Despite these possibilities, we still found considerable lineage sharing between these two species. Tawny and Berthelot’s pipits diverged only ca. 2.5 Ma ago (Chapter 2; Voelker 1999); the lineage sharing could therefore be attributed to incomplete lineage sorting (Klein et al. 1993, 1998). Another possibility is that trans-species persistence of MHC lineages has been promoted by balancing selection, whereby allele lineages that confer selective advantage persist over evolutionary time with little change (Klein et al. 1993). Such trans-species persistence has been reported in many MHC studies (Graser et al. 1996; Richardson and Westerdahl 2003; Jaratlerdsiri et al. 2014). Interestingly, we have previously found that the two pipit species also share a small number of alleles at Toll-like receptors (3 out of 94 alleles across five loci, González-Quevedo et al., The role of drift and selection in shaping variation at innate immune genes in oceanic island populations, unpublished). That we see similar patterns in the two immune gene families may suggest a greater role for incomplete lineage sorting than pathogen-mediated selection in the two species sharing alleles, but testing this hypothesis would require both a larger sample of tawny pipits and assessment of other loci.

Pathogen-mediated selection on the PBR is thought to be the main force maintaining variation at MHC (Westerdahl et al. 2005; Evans and Neff 2009; Spurgin and Richardson 2010). In Berthelot’s pipits MHC class I alleles, we found no evidence of an elevated *d*_N_/*d*_S_ in the PBR compared to the non-PBR which is the classic indication of selection at MHC (Hughes and Nei 1988; Bernatchez and Landry 2003). However, we found that both *d*_N_ and *d*_S_ were significantly higher at the PBR than at the non PBR (Fig. 4), which is to be expected when gene conversion is the main source of variation (Ohta 1995). Gene conversion results in the transfer of sections of DNA containing synonymous and non-synonymous changes between alleles within or across loci. When this process involves sites in the PBR, the new molecular conformation of amino acids encoded for may be advantageous if it creates a new allele that allows the better binding of peptides from pathogens present within the population (Ohta 1995). On the other hand, if such events occur within the non-PBR they are likely to be selected against, because this region is functionally constrained due to its role in molecule integrity (Klein et al. 1993). Several previous studies have argued that gene conversion is one of the main mutational forces generating MHC allelic variation in vertebrates (reviewed in Hogstrand and Bohme 1999; Ohta 1999), a hypothesis that has been directly supported by earlier data from Berthelot’s pipit (Spurgin et al. 2011). In line with this earlier study, we also detected four gene conversion events and an elevated recombination to mutation ratio across the MHC alleles of both pipit species (1.4 for Berthelot’s pipit and 2.0 for tawny pipit alleles). Within the exon amplified, we found evidence of historical positive selection at ten specific amino acid sites, of which four corresponded to the estimated PBR. Three of these sites have previously been identified to be under positive selection in other bird species (Sutton et al. 2013), which suggests that these sites are important determinants of the binding properties of the PBR in avian species.

Overall, our results suggest that the Berthelot’s pipit population on Tenerife has reduced allelic diversity at the MHC compared to its closest sister species. Nevertheless, the allele lineages that persisted, or were generated after the colonization of Tenerife, display divergent antigen binding properties. These divergent alleles might be sufficient to successfully initiate an adequate immune response to the local pathogens that threaten this population. This mechanism can have significant implications for the survival and establishment of populations that colonize new areas possibly containing novel diseases. Berthelot’s pipits in Tenerife have a high incidence of malaria (Gonzalez-Quevedo et al. 2014) and the MHC is likely to play a role in the epidemiology of this disease in the population given that it has previously been linked to malaria resistance (Hill et al. 1991; Westerdahl et al. 2005; Bonneaud et al. 2006). This screening methodology and the data outlined in the present study can now be used to assess the association of MHC alleles and disease susceptibility/resistance, and to investigate what causes temporal changes in MHC allele frequency within populations to further understand how pathogen mediated selection might shape the variation of the MHC at the population level.

## Electronic supplementary material

Below is the link to the electronic supplementary material.

ESM 1 (DOCX 720 kb)

## References

[CR1] 454 Life Sciences Corp (2009) Using Multiplex Identifier (MID) Adaptors for the GS FLX titanium chemistry - extended MID Set 454 sequencing technical bulletin 005–2009:1–7

[CR2] Alcaide M, Edwards SV, Negro JJ, Serrano D, Tella JL (2008). Extensive polymorphism and geographical variation at a positively selected MHC class IIB gene of the lesser kestrel (*Falco naumanni*). Mol Ecol.

[CR3] Alcaide M, Liu M, Edwards SV (2013). Major histocompatibility complex class I evolution in songbirds: universal primers, rapid evolution and base compositional shifts in exon 3. Peer J.

[CR4] Babik W (2010). Methods for MHC genotyping in non-model vertebrates. Mol Ecol Resour.

[CR5] Babik W, Taberlet P, Ejsmond MJ, Radwan J (2009). New generation sequencers as a tool for genotyping of highly polymorphic multilocus MHC system. Mol Ecol Resour.

[CR6] Baquero JE (2006). Reference strand conformational analysis (RSCA) is a valuable tool in identifying MHC-DRB sequences in three species of *Aotus* monkeys. Immunogenetics.

[CR7] Bernatchez L, Landry C (2003). MHC studies in nonmodel vertebrates: what have we learned about natural selection in 15 years?. J Evol Biol.

[CR8] Beuf KD, Schrijver JD, Thas O, Criekinge WV, Irizarry RA, Clement L (2012). Improved base-calling and quality scores for 454 sequencing based on a Hurdle Poisson model. BMC Bioinforma.

[CR9] Bjorkman PJ, Saper MA, Samraoui B, Bennett WS, Strominger JL, Wiley DC (1987). Structure of the human class-I histocompatibility antigen, HLA-A2. Nature.

[CR10] Bodmer JG (1997). Nomenclature for factors of the HLA system, 1996. Tissue Antigens.

[CR11] Bollmer JL, Hull JM, Ernest HB, Sarasola JH, Parker PG (2011). Reduced MHC and neutral variation in the Galapagos hawk, an island endemic. BMC Evol Biol.

[CR12] Boni MF, Posada D, Feldman MW (2007). An exact nonparametric method for inferring mosaic structure in sequence triplets. Genetics.

[CR13] Bonneaud C, Perez-Tris J, Federici P, Chastel O, Sorci G (2006). Major histocompatibility alleles associated with local resistance to malaria in a passerine. Evolution.

[CR14] Bradley RD, Hillis DM (1997). Recombinant DNA sequences generated by PCR amplification. Mol Biol Evol.

[CR15] Brown JH, Jardetzky TS, Gorga JC, Stern LJ, Urban RG, Strominger JL, Wiley DC (1993). 3-Dimensional structure of the human class II-histocompatinility antigen HLA-DR1. Nature.

[CR16] Bryant D, Moulton V (2004). Neighbor-Net: an agglomerative method for the construction of phylogenetic networks. Mol Biol Evol.

[CR17] Chelvanayagam G (1996). A roadmap for HLA-A, HLA-B, and HLA-C peptide binding specificities. Immunogenetics.

[CR18] Cheng Y (2012). Antigen-presenting genes and genomic copy number variations in the Tasmanian devil MHC. BMC Genomics.

[CR19] Cramp S, Perrins CM (1977–1994) Handbook of the birds of Europe, the Middle East and Africa. The birds of the western Palearctic. Oxford University Press, Oxford

[CR20] Doytchinova IA, Flower DR (2005). In silico identification of supertypes for class II MHCs. J Immunol.

[CR21] Dunn PO, Bollmer JL, Freeman-Gallant CR, Whittingham LA (2013). MHC variation is related to a sexually selected ornament, survival, and parasite resistance in common yellowthroats. Evolution.

[CR22] Edwards SV, Hedrick PW (1998). Evolution and ecology of MHC molecules: from genomics to sexual selection. Trends Ecol Evol.

[CR23] Ellison A, Allainguillaume J, Girdwood S, Pachebat J, Peat KM, Wright P, Consuegra S (2012). Maintaining functional major histocompatibility complex diversity under inbreeding: the case of a selfing vertebrate. Proc R Soc B Biol Sci.

[CR24] Evans ML, Neff BD (2009). Major histocompatibility complex heterozygote advantage and widespread bacterial infections in populations of Chinook salmon (*Oncorhynchus tshawytscha*). Mol Ecol.

[CR25] Frank SA (2002). Immunology and evolution of infectious disease.

[CR26] Galan M, Guivier E, Caraux G, Charbonnel N, Cosson JF (2010). A 454 multiplex sequencing method for rapid and reliable genotyping of highly polymorphic genes in large-scale studies. BMC Genomics.

[CR27] Garcia-Del-Rey E, Cresswell W (2007). The breeding biology of the endemic Berthelot's Pipit, *Anthus berthelotii*, in a harsh oceanic island environment (Tenerife, Canary Islands). Ostrich.

[CR28] Geliebter J, Nathenson SG (1988). Microrecombinations generate sequence diversity in the murine major histocompatibility complex: analysis of the Kbm3, Kbm4, Kbm10, and Kbm11 mutants. Mol Cell Biol.

[CR29] Gibbs MJ, Armstrong JS, Gibbs AJ (2000). Sister-scanning: a Monte Carlo procedure for assessing signals in recombinant sequences. Bioinformatics.

[CR30] Gonzalez-Quevedo C, Davies RG, Richardson DS (2014). Predictors of malaria infection in a wild bird population: landscape-level analyses reveal climatic and anthropogenic factors. J Anim Ecol.

[CR31] Graser R, O'Huigin C, Vincek V, Meyer A, Klein J (1996). Trans-species polymorphism of class II Mhc loci in *Danio* fishes. Immunogenetics.

[CR32] Hill AVS (1991). Common West African HLA antigens are associated with protection from severe malaria. Nature.

[CR33] Hogstrand K, Bohme J (1999). Gene conversion can create new MHC alleles. Immunol Rev.

[CR34] Holcomb CL, Rastrou M, Williams TC, Goodridge D, Lazaro AM, Tilanus M, Erlich HA (2014). Next-generation sequencing can reveal in vitro-generated PCR crossover products: some artifactual sequences correspond to HLA alleles in the IMGT/HLA database. Tissue Antigens.

[CR35] Huchard E, Weill M, Cowlishaw G, Raymond M, Knapp LA (2008). Polymorphism, haplotype composition, and selection in the Mhc-DRB of wild baboons. Immunogenetics.

[CR36] Hudson RR, Kaplan NL (1985). Statistical properties of the number of recombination events in the history of a sample of DNA sequences. Genetics.

[CR37] Hughes AL, Nei M (1988). Pattern of nucleotide substitution at major histocompatibility complex class-I loci reveals overdominant selection. Nature.

[CR38] Huse SM, Huber JA, Morrison HG, Sogin ML, Mark Welch D (2007). Accuracy and quality of massively parallel DNA pyrosequencing. Genome Biol.

[CR39] Huson DH, Bryant D (2006). Application of phylogenetic networks in evolutionary studies. Mol Biol Evol.

[CR40] Illera JC, Lorenzo JA (2007). Bisbita Caminero *Anthus berthelotii*. Atlas de las aves nidificantes en el archipiélago Canario (1997–2003).

[CR41] Illera JC, Emerson BC, Richardson DS (2007). Population history of Berthelot’s pipit: colonization, gene flow and morphological divergence in Macaronesia. Mol Ecol.

[CR42] Jaratlerdsiri W (2014). Evolution of MHC class I in the Order Crocodylia. Immunogenetics.

[CR43] Jarvi SI, Tarr CL, McIntosh CE, Atkinson CT, Fleischer RC (2004). Natural selection of the major histocompatibility complex (MHC) in Hawaiian honeycreepers (Drepanidinae). Mol Ecol.

[CR44] Jombart T (2008). Adegenet: a R package for the multivariate analysis of genetic markers. Bioinformatics.

[CR45] Jombart T, Devillard S, Balloux F (2010). Discriminant analysis of principal components: a new method for the analysis of genetically structured populations. BMC Genet.

[CR46] Kelley J, Walter L, Trowsdale J (2005). Comparative genomics of major histocompatibility complexes. Immunogenetics.

[CR47] Kiemnec-Tyburczy KM, Richmond JQ, Savage AE, Zamudio KR (2010). Selection, trans-species polymorphism, and locus identification of major histocompatibility complex class II beta alleles of New World ranid frogs. Immunogenetics.

[CR48] Klein J, Satta Y, Ohuigin C, Takahata N (1993). The molecular descent of the major histocompatibility complex. Annu Rev Immunol.

[CR49] Klein J, O'Huigin C, Mims CA, Lines J, Deutsch J, Hughes AL (1994). MHC polymorphism and parasites (and discussion). Phil Trans R Soc Lond B Biol Sci.

[CR50] Klein J, Sato A, Nagl S, O'Huigin C (1998). Molecular trans-species polymorphism. Annu Rev Ecol Syst.

[CR51] Kloch A, Babik W, Bajer A, Sinski E, Radwan J (2010). Effects of an MHC-DRB genotype and allele number on the load of gut parasites in the bank vole *Myodes glareolus*. Mol Ecol.

[CR52] Lenz TL, Becker S (2008). Simple approach to reduce PCR artefact formation leads to reliable genotyping of MHC and other highly polymorphic loci—implications for evolutionary analysis. Gene.

[CR53] Librado P, Rozas J (2009). DnaSP v5: a software for comprehensive analysis of DNA polymorphism data. Bioinformatics.

[CR54] Lighten J, van Oosterhout C, Bentzen P (2014). Critical review of NGS analyses for *de novo* genotyping multigene families. Mol Ecol.

[CR55] Lighten J, van Oosterhout C, Paterson IG, McMullan M, Bentzen P (2014). Ultra-deep Illumina sequencing accurately identifies MHC class IIb alleles and provides evidence for copy number variation in the guppy (*Poecilia reticulata*). Mol Ecol Resour.

[CR56] Margulies M (2005). Genome sequencing in microfabricated high-density picolitre reactors. Nature.

[CR57] Martin DP, Lemey P, Lott M, Moulton V, Posada D, Lefeuvre P (2010). RDP3: a flexible and fast computer program for analyzing recombination. Bioinformatics.

[CR58] May S, Zeisset I, Beebee TJC (2011). Larval fitness and immunogenetic diversity in chytrid-infected and uninfected natterjack toad (*Bufo calamita*) populations. Conserv Genet.

[CR59] Meyerhans A, Vartanian JP, Wainhobson S (1990). DNA recombinatiion during PCR. Nucleic Acids Res.

[CR60] Meyer-Lucht Y, Otten C, Puettker T, Pardini R, Paul Metzger J, Sommer S (2010). Variety matters: adaptive genetic diversity and parasite load in two mouse opossums from the Brazilian Atlantic forest. Conserv Genet.

[CR61] Mona S (2008). Disentangling the effects of recombination, selection, and demography on the genetic variation at a major histocompatibility complex class II gene in the alpine chamois. Mol Ecol.

[CR62] Murrell B, Wertheim JO, Moola S, Weighill T, Scheffler K, Pond SLK (2012). Detecting individual sites subject to episodic diversifying selection. PLoS Genet.

[CR63] Murrell B, Moola S, Mabona A, Weighill T, Sheward D, Pond SLK, Scheffler K (2013). FUBAR: a fast, unconstrained bayesian approximation for inferring selection. Mol Biol Evol.

[CR64] Mwenda JM, Hashiba K, Bambra CS, Shotake T (1997). Analysis of primate major histocompatibility complex (MHC)-DQA1 locus by PCR-single strand conformation polymorphism (SSCP). Cell Mol Biol.

[CR65] Nei M, Gojobori T (1986). Simple methods for estimating the numbers of synonymous and nonsynonymous nucleotide substitutions. Mol Biol Evol.

[CR66] Niskanen AK (2014). Balancing selection and heterozygote advantage in major histocompatibility complex loci of the bottlenecked Finnish wolf population. Mol Ecol.

[CR67] Ohta T (1995). Gene conversion vs. point mutation in generating variability at the antigen recognition site of major histocompatibility complex loci. J Mol Evol.

[CR68] Ohta T (1999). Effect of gene conversion on polymorphic patterns at major histocompatibility complex loci. Immunol Rev.

[CR69] Padidam M, Sawyer S, Fauquet CM (1999). Possible emergence of new geminiviruses by frequent recombination. Virology.

[CR70] Pond SLK, Frost SDW (2005). Datamonkey: rapid detection of selective pressure on individual sites of codon alignments. Bioinformatics.

[CR71] Pond SLK, Frost SDW (2005). Not so different after all: a comparison of methods for detecting amino acid sites under selection. Mol Biol Evol.

[CR72] Posada D, Crandall KA (2001). Evaluation of methods for detecting recombination from DNA sequences: computer simulations. Proc Natl Acad Sci U S A.

[CR73] Potts WK, Wakeland EK (1990). Evolution of diversity at the major histocompatibility complex. Trends Ecol Evol.

[CR74] Promerová M, Babik W, Bryja J, Albrecht T, Stuglik M, Radwan J (2012). Evaluation of two approaches to genotyping major histocompatibility complex class I in a passerine-CE-SSCP and 454 pyrosequencing. Mol Ecol Resour.

[CR75] R Development Core Team (2011). R: a language and environment for statistical computing.

[CR76] Radwan J, Biedrzycka A, Babik W (2010). Does reduced MHC diversity decrease viability of vertebrate populations?. Biol Conserv.

[CR77] Richardson DS, Westerdahl H (2003). MHC diversity in two *Acrocephalus* species: the outbred Great reed warbler and the inbred Seychelles warbler. Mol Ecol.

[CR78] Richardson DS, Jury FL, Blaakmeer K, Komdeur J, Burke T (2001). Parentage assignment and extra-group paternity in a cooperative breeder: the Seychelles warbler (*Acrocephalus sechellensis*). Mol Ecol.

[CR79] Robinson J, Halliwell JA, McWilliam H, Lopez R, Parham P, Marsh SGE (2013). The IMGT/HLA database. Nucleic Acids Res.

[CR80] Sandberg M, Eriksson L, Jonsson J, Sjostrom M, Wold S (1998). New chemical descriptors relevant for the design of biologically active peptides. A multivariate characterization of 87 amino acids. J Med Chem.

[CR81] Schwensow N, Fietz J, Dausmann KH, Sommer S (2007). Neutral versus adaptive genetic variation in parasite resistance: importance of major histocompatibility complex supertypes in a free-ranging primate. Heredity.

[CR82] Sepil I, Moghadam HK, Huchard E, Sheldon BC (2012). Characterization and 454 pyrosequencing of Major Histocompatibility Complex class I genes in the great tit reveal complexity in a passerine system. BMC Evol Biol.

[CR83] Sepil I, Lachish S, Hinks AE, Sheldon BC (2013) Mhc supertypes confer both qualitative and quantitative resistance to avian malaria infections in a wild bird population. Proc R Soc B Biol Sci 280 10.1098/rspb.2013.0134 PMC3619505 23516242

[CR84] Sidney J (1995). Several HLA alleles share overlapping peptide specificities. J Immunol.

[CR85] Smith JM (1992). Analyzing the mosaic structure of genes. J Mol Evol.

[CR86] Sommer S (2005). The importance of immune gene variability (MHC) in evolutionary ecology and conservation. Front Zool.

[CR87] Sommer S, Courtiol A, Mazzoni CJ (2013). MHC genotyping of non-model organisms using next-generation sequencing: a new methodology to deal with artefacts and allelic dropout. BMC Genomics.

[CR88] Spurgin LG, Richardson DS (2010). How pathogens drive genetic diversity: MHC, mechanisms and misunderstandings. Proc R Soc B Biol Sci.

[CR89] Spurgin LG, van Oosterhout C, Illera JC, Bridgett S, Gharbi K, Emerson BC, Richardson DS (2011). Gene conversion rapidly generates major histocompatibility complex diversity in recently founded bird populations. Mol Ecol.

[CR90] Spurgin LG, Illera JC, Padilla DP, Richardson DS (2012). Biogeographical patterns and co-occurrence of pathogenic infection across island populations of Berthelot's pipit (*Anthus berthelotii*). Oecologia.

[CR91] Spurgin LG, Illera JC, Jorgensen TH, Dawson DA, Richardson DS (2014). Genetic and phenotypic divergence in an island bird: isolation by distance, by colonisation or by adaptation?. Mol Ecol.

[CR92] Sutton JT, Robertson BC, Grueber CE, Stanton JAL, Jamieson IG (2013). Characterization of MHC class II B polymorphism in bottlenecked New Zealand saddlebacks reveals low levels of genetic diversity. Immunogenetics.

[CR93] Tamura K, Peterson D, Peterson N, Stecher G, Nei M, Kumar S (2011). MEGA5: molecular evolutionary genetics analysis using maximum likelihood, evolutionary distance, and maximum parsimony methods. Mol Biol Evol.

[CR94] Torimiro JN (2006). HLA class I diversity among rural rainforest inhabitants in Cameroon: identification of A*2612-B*4407 haplotype. Tissue Antigens.

[CR95] Ujvari B, Belov K (2011). Major histocompatibility complex (MHC) markers in conservation biology. Int J Mol Sci.

[CR96] van Oosterhout C (2009). Trans-species polymorphism, HLA-disease associations and the evolution of the MHC. Commun Integr Biol.

[CR97] van Oosterhout C (2013). Maintenance of major histocompatibility supertype variation in selfing vertebrate is no evidence for overdominant selection. Proc R Soc B Biol Sci.

[CR98] Voelker G (1999). Dispersal, vicariance, and clocks: historical biogeography and speciation in a cosmopolitan passerine genus (*Anthus*: motacillidae). Evolution.

[CR99] Wakelin D (1996). Immunity to parasites: how parasitic infections are controlled.

[CR100] Wakelin D, Apanius V (1997). Immune defence: genetic control. Host-parasite evolution, general principles and avian models.

[CR101] Westerdahl H, Hansson B, Bensch S, Hasselquist D (2004). Between-year variation of MHC allele frequencies in great reed warblers: selection or drift?. J Evol Biol.

[CR102] Westerdahl H, Waldenstrom J, Hansson B, Hasselquist D, von Schantz T, Bensch S (2005). Associations between malaria and MHC genes in a migratory songbird. Proc R Soc B Biol Sci.

[CR103] Worley K, Gillingham M, Jensen P, Kennedy LJ, Pizzari T, Kaufman J, Richardson DS (2008). Single locus typing of MHC class I and class IIB loci in a population of red jungle fowl. Immunogenetics.

[CR104] Wright DJ, Spurgin LG, Collar NJ, Komdeur J, Burke T, Richardson DS (2014). The impact of translocations on neutral and functional genetic diversity within and among populations of the Seychelles warbler. Mol Ecol.

[CR105] Yasukochi Y, Kurosaki T, Yoneda M, Koike H, Satta Y (2012). MHC class II DQB diversity in the Japanese black bear, *Ursus thibetanus japonicus*. BMC Evol Biol.

[CR106] Zagalska-Neubauer M, Babik W, Stuglik M, Gustafsson L, Cichon M, Radwan J (2010). 454 sequencing reveals extreme complexity of the class II Major Histocompatibility Complex in the collared flycatcher. BMC Evol Biol.

