# Supplementary material for: 454 screening of individual MHC variation in an endemic island passerine
Source: Immunogenetics. 2014 Dec 18;67(3):149–62. doi: 10.1007/s00251-014-0822-1 (PMC4325181; doi:10.1007/s00251-014-0822-1)

**Supplementary Information**

The following information is available for this article online.

**Appendix 1. Description of workflow used to identify chimeras within amplicons.**

To test whether a given sequence Z could be a chimera of sequences X and Y, we listed the base pair positions at which Z differed from each of the putative parent sequences. If the first difference from one parent occurred after the last difference from the other (or vice versa), sequence Z was listed as a chimera. This is because such a pattern means that sequence Z is identical to one parent sequence before a given point and identical to the other parent sequence after that point.

In accordance with the rationale of Sommer *et* al’s (2013) workflow, we only tested whether a given sequence could be a chimera of two more frequent sequences within the amplicon of interest. For computational efficiency, we restricted the list of putative parent sequences for chimeras to the top twenty most frequent sequences in an amplicon.

**Table S1** Number of clusters classified as chimera, 1-2 bp difference, >2 bp difference, putative artefact, putative allele and unclassified variant in each of the three bioinformatics steps to classify reads from Berthelot’s pipits MHC class I exon 3 sequences. In step 1, variants are classified as putative alleles if they are the most common sequence in an amplicon, and as artefacts if they are singletons. The remaining clusters are classified as chimeras, 1-2 bp difference or >2 bp difference compared to the most similar cluster. Variants in each of these three classes are subject to steps 2 and 3 and further classified as putative artefact, putative allele or unclassified variant. Note that alleles can be identified in different bioinformatic steps in each individual.

|  | Step 1 | Step 2 | | | Step 3 | | |
| --- | --- | --- | --- | --- | --- | --- | --- |
| Variant class |  | chimera | 1-2 bp diff | >2 bp diff | chimera | 1-2 bp diff | >2 bp diff |
| chimera | 2,023 |  |  |  |  |  |  |
| 1-2 bp difference | 24,631 |  |  |  |  |  |  |
| >2 bp difference | 14,583 |  |  |  |  |  |  |
| Putative artefact | 17,408 | 1,903 | 21,960 | 3,863 |  |  | 5,800 |
| Putative allele | 640 |  |  |  | 29 | 51 | 611 |
| Unclassified |  |  |  |  | 91 | 2,620 | 4,309 |

**Figure S1** Fusion primers used for preparation of MHC class I exon 3 amplicons. Nine forward and nine reverse primers were used in combination to identify each of 80 amplicons.

Reverse primers:

F1=DG2-MID1: 5’- CGTATCGCCTCCCTCGCGCCATCAGACGAGTGCGTTTGCGCTCYAGCTCYTTCTGCT -3’
F2=DG2-MID2: 5’- CGTATCGCCTCCCTCGCGCCATCAGACGCTCGACATTGCGCTCYAGCTCYTTCTGCT -3’
F3=DG2-MID3: 5’- CGTATCGCCTCCCTCGCGCCATCAGAGACGCACTCTTGCGCTCYAGCTCYTTCTGCT -3’
F4=DG2-MID4: 5’- CGTATCGCCTCCCTCGCGCCATCAGAGCACTGTAGTTGCGCTCYAGCTCYTTCTGCT-3’
F5=DG2-MID5: 5’- CGTATCGCCTCCCTCGCGCCATCAGATCAGACACGTTGCGCTCYAGCTCYTTCTGCT -3’
F6=DG2-MID7: 5’- CGTATCGCCTCCCTCGCGCCATCAGCGTGTCTCTATTGCGCTCYAGCTCYTTCTGCT -3’
F7=DG2-MID8: 5’- CGTATCGCCTCCCTCGCGCCATCAGCTCGCGTGTCTTGCGCTCYAGCTCYTTCTGCT -3’

F8=DG2-MID10: 5’- CGTATCGCCTCCCTCGCGCCATCAGTCTCTATGCGTTGCGCTCYAGCTCYTTCTGCT -3’

F9=DG2-MID11: 5’- CGTATCGCCTCCCTCGCGCCATCAGTGATACGTCTTTGCGCTCYAGCTCYTTCTGCT -3’

Reverse primers:
R1=GENDG-MID1: 5’- CTATGCGCCTTGCCAGCCCGCTCAGACGAGTGCGTTCCCCACAGGTCTCCACAC -3’
R2=GENDG-MID2: 5’- CTATGCGCCTTGCCAGCCCGCTCAGACGCTCGACATCCCCACAGGTCTCCACAC-3’
R3=GENDG-MID3: 5’- CTATGCGCCTTGCCAGCCCGCTCAGAGACGCACTCTCCCCACAGGTCTCCACAC -3’
R4=GENDG-MID4: 5’- CTATGCGCCTTGCCAGCCCGCTCAGAGCACTGTAGTCCCCACAGGTCTCCACAC -3’
R5=GENDG-MID5: 5’- CTATGCGCCTTGCCAGCCCGCTCAGATCAGACACGTCCCCACAGGTCTCCACAC -3’
R6=GENDG-MID7: 5’- CTATGCGCCTTGCCAGCCCGCTCAGCGTGTCTCTATCCCCACAGGTCTCCACAC -3’
R7=GENDG-MID8: 5’- CTATGCGCCTTGCCAGCCCGCTCAGCTCGCGTGTCTCCCCACAGGTCTCCACAC -3’
R8=GENDG-MID10: 5’- CTATGCGCCTTGCCAGCCCGCTCAGTCTCTATGCGTCCCCACAGGTCTCCACAC -3’
R9=GENDG-MID11: 5’- CTATGCGCCTTGCCAGCCCGCTCAGTGATACGTCTTCCCCACAGGTCTCCACAC -3’

**Figure S2** Frequencies of unclassified variants identified after the bioinformatics processing of MHC class I exon 3 sequences obtained from 310 Berthelot’s pipits (*Anthus berthelotii*) and 10 tawny pipits (*A. campestris*). For simplicity only the unclassified variants found in four or more samples are shown. The two most common unclassified variants matched two alleles previously described in Berthelot’s pipits ([Spurgin *et al.* 2011](#_ENREF_88)), ANBE3 and ANBE31, detected in both replicates of 260 and 253 Berthelot’s pipit samples, respectively


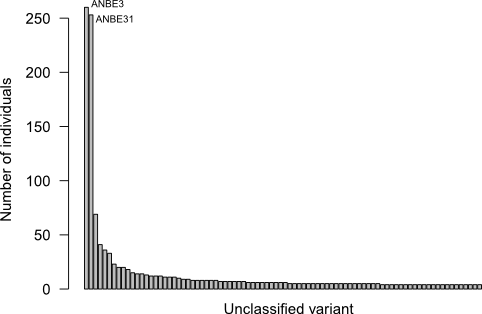


**Figure S3** Mean intra-amplicon frequencies for each of the MHC class I exon 3 alleles identified in a) Berthelot’s pipits (*Anthus berthelotii*)) and b) tawny pipits (*A. campestris*). Error bars are standard errors.


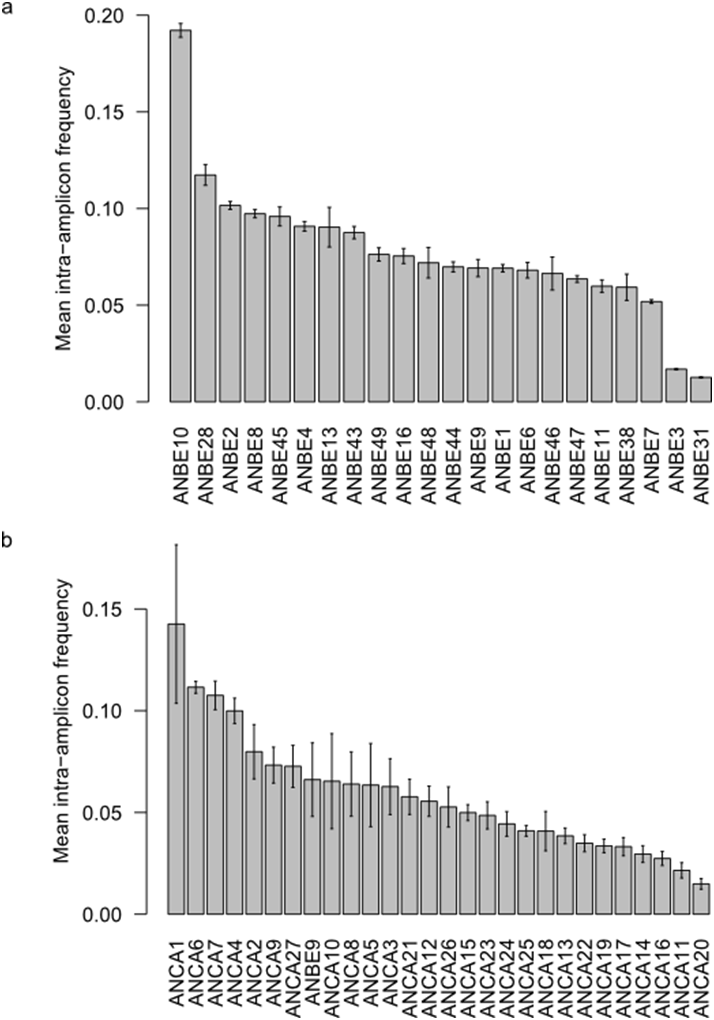


**Figure S4** Number of Berthelot’s pipit, *Anthus berthelotii* (upper panel) or tawny pipit, *A. campestris* (lower panel) MHC class I exon 3 alleles (at the nucleotide level) per individual in the sampled population.


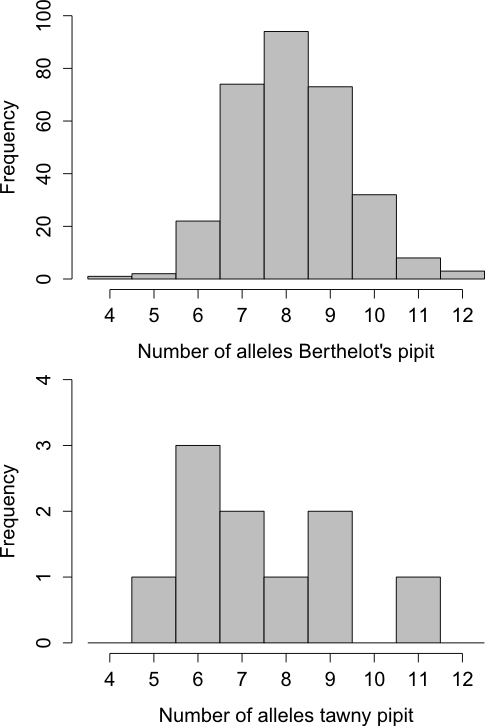


**Figure S5** Standardised amplification efficiency of MHC class I exon 3 alleles identified in a) 310 Berthelot’s pipits from Tenerife, and b) 10 tawny pipits. The horizontal line represents the amplification efficiency of 1.0, obtained for the reference allele ANBE11 (Berthelot’s pipits) or ANCA17 (tawny pipits).


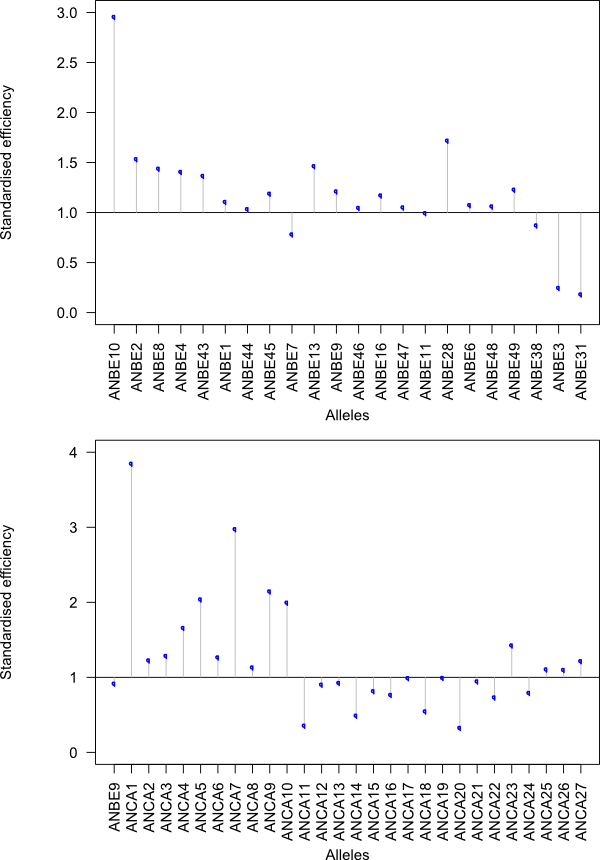


**Figure S6** Amino acid alignment of the 20 Berthelot’s pipit (*Anthus berthelotii*) and 23 tawny pipit (*A. campestris*) functional MHC class I exon 3 alleles identified in this study. Dots in the alignment denote consensus amino acids with ANBE1. The amino acids comprising the peptide binding region (PBR) are shown with asterisks. Amino acids identified as being under positive selection using Berthelot’s and tawny pipit alleles are indicated with a plus sign.


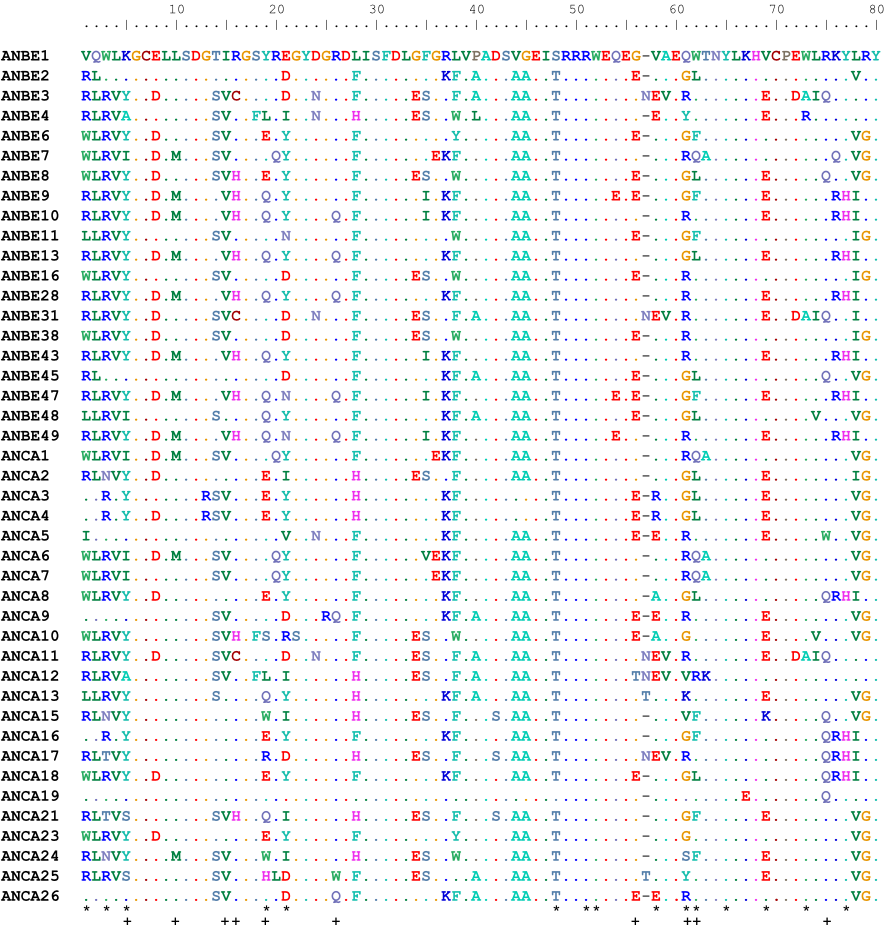


**Figure S7** Alignment of amino acids of peptide binding region (PBR) sequences of a) Berthelot’s pipit (*Anthus berthelotii*) or b) tawny pipit (*Anthus campestris*) MHC class I, exon 3 alleles. Squares enclose similar PBR sequences. Consensus with first sequence is denoted by dots. Order of amino acids corresponds to the position of PBR amino acids in the full exon, as shown in figure 6.


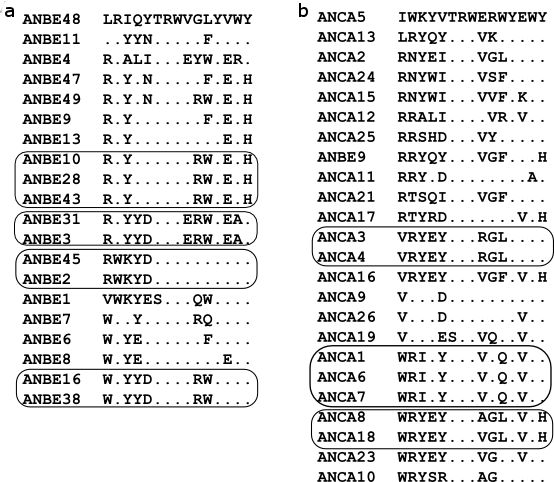

Supplement: Supplementary file 1 — (DOCX 720 kb) [file 251_2014_822_MOESM1_ESM.docx]
